# The Association of Long-Term Use of Proton Pump Inhibitors and Histamine H_2_ Receptor Antagonists with Clinical Complications in Patients with Severe Sepsis

**DOI:** 10.1155/2022/4093595

**Published:** 2022-06-28

**Authors:** Jiamin Gao, Senlin Ma, Siyuan Xu, Mingquan Chen

**Affiliations:** Department of Emergency, Huashan Hospital, Fudan University, Shanghai, China

## Abstract

**Objective:**

Proton pump inhibitors (PPIs) are commonly used to treat gastric acidity, and their frequent use may trigger various malfunctioning, such as cardiac, renal, and liver function failure. In the current study, we evaluated the association between the excessive use of the PPIs and the clinical complications of intensive care unit (ICU) septic patients.

**Methods:**

A total of 208188 patients were analyzed from 2016 to 2017 through the China Critical Care Sepsis Trial (CCCST) database. The characteristics of the study group and outcome of events from the PPI- and H_2_ blocker-using groups were reported. To get unbiased results, the data from the target trials were randomly assigned for PPI and H_2_ blocker groups.

**Result:**

The data revealed 43.34 excess deaths (95% confidence intensive (CI) 25.12 to 62.02) per 1000 patients in patients extensively consuming PPI drugs. The sepsis with chronic kidney disease attributed to deaths 21.36; 95% CI (9.34 to 23.23). However, comorbidities, including circulatory diseases (16.34; 95% CI 5.78 to 23.45), nervous system (2.08; 95% CI 1.56 to 6.34), mental disorders (1.87; 95% CI 1.65 to 2.95), genitourinary system (5.23; 95% CI 3.69 to 8.89), and infectious and parasitic disease (4.17; 95% CI 1.44 to 7.49), were also reported. Extensive use of the PPIs and H_2_ blockers was associated with esophageal adenocarcinoma, Barrett's esophagus, neoplasms, and GI cancers.

**Conclusion:**

We conclude that the excessive use of PPI in sepsis patients triggers chronic kidney disease which has a higher clinical complication rate among others.

## 1. Introduction

The proton pump inhibitors are medicines used for the acid repression in peptic ulcer (PU) as well as controlling gastroesophageal reflux disease (GERD) [1]. Multiple PPIs such as lansoprazole in 1995, pantoprazole in 1997, rabeprazole in 1999, and the S-enantiomer of omeprazole as well as esomeprazole in 2001 were developed [2, 3]. The PPIs are not the ideal antiacid secretory drugs [4], and recently, new compounds have been developed which have extended acid suppression activity [5, 6]. Once the patients are prescribed with PPI, they tend to stay on the same treatment for a longer time [7] or permanently in older patients [8]. The gastric parietal cells were proved to be effective in the suppression of acid secretion [9]. PPIs are used in both treatment and the prevention of gastric and duodenal ulcers and gastroesophageal reflux disease and in the eradication of Helicobacter pylori. Their ubiquitous use is also due to the administration of PPI to patients receiving nonsteroidal anti-inflammatory drugs or antiplatelet agents [10, 11]. In addition to the well-known use in treatment of inflammation of the upper gastrointestinal tract, the number of alternative PPI consumption is constantly increasing, including the treatment of a variety of respiratory symptoms, sleep disorders, and hypersensitivity and hyperactivity in children [12].

As a category of well-tolerated drugs, PPIs are not devoid of side effects. Different adverse reactions have been detected in the patients, specifically individuals with bacterial infection and sepsis. Since the early 80s, the increased risks of infections were found among patients extensively taking antacids [1, 2, 13, 14]. Such patients with any intercurrent condition are at much higher risk of developing sepsis along with other organ failures, most likely renal failure [15]. The prevalence of chronic kidney disease (CKD) increases, and excessive consumption of the PPIs could be one of the potential risk factors, potentially moderated by recurrent acute kidney injury [16].

Severe sepsis or septic shock is the very critical stage of the disease leading to multiple organ failure and mortality in various populations [17]. The “Surviving Sepsis Campaign (SSC)” proposed guidelines that direct evidence to regulate patients suffering from sepsis and enhance their consequences [18, 19]. Even the SSC guidelines suggest the usage of PPIs over histamine-2-receptor antagonists (H2RAs) in critical septic ulcer patients [20, 21].

On the other hand, the H_2_ blockers are mainly used for duodenal ulcer and gastric ulcer [22]. After 6~8 weeks, the healing rate is high. Prolonging the medication can reduce the recurrence. Zollinger Ellison syndrome requires a large dose. Other diseases with excessive gastric acid secretion, such as gastrointestinal anastomotic ulcer, reflux esophagitis, and bleeding caused by peptic ulcer and acute gastritis, can also be used. H_2_ receptor blockers mainly inhibit basal gastric acid and nocturnal gastric acid secretion by blocking H_2_ receptors in gastric parietal cells. At the same time, they also inhibit gastric acid secretion caused by gastrin and M receptor agonists. The main H_2_ receptor blockers are cimetidine, ranitidine, famotidine, nizatidine, rosatidine, and newly marketed ethylbromotidine and miphenetidine.

Long-term use of H_2_ blockers or proton pump inhibitors (PPI) was reported to be a marker for increased risk of EAC. This may be due to the underlying disease. In fact, PPI use seems to lower the risk of dysplasia in BE. With the recent concerns raised about harmful consequences of chronic PPI use, there is renewed interest about other means of acid suppression, based on the fact that severe sepsis always causes other abnormalities such as organ failure leading to death. However, the relationship between the antacid or proton pump inhibitors (PPIs) in gastric ulcer or sepsis patients with chronic kidney disease and mortality rate has not been established. Therefore, we conducted a large-scale study to find out the association between the excessive use of the PPIs and H_2_ blockers and the mortality rate in sepsis patients at the ICU with chronic kidney disease and other associated abnormalities.

## 2. Methods

### 2.1. Study Design

Targeted randomized controlled trial data were analyzed for consumption of PPIs and H_2_ blockers in patients with gastric sepsis for different clinical parameters and mortality. Casual inference strategies were linked to estimate the mortality associated with the use of PPI and H_2_ blockers.

Patients with gastric acidity using different PPI and antacids were recruited from 1 August 2016 to 31 July 2017 and then followed up for 3 years to analyze the associations between proton pump inhibitors (PPI), H_2_ blockers, and causes of death. All patients agreed to the informed consent.

### 2.2. Inclusion and Exclusion Criteria

PPI inclusion criteria: (1) only patients administered PPI for more than 90 days in total past 180 days were selected for the study. (2) These candidates were screened out based on dual consumption of either H_2_ blockers or PPI in the past 180 days. (3) Finally, pure PPI users admitted in the ICU were selected.

H_2_ inclusion criteria: (1) the patients with H_2_ blocker use in 90 days were selected for the study. (2) Patients used mix either PPI or H_2_ blocker in 90 days were excluded from the study. (3) Finally, patients with pure use of H_2_ blockers admitted into the ICU were excluded.

### 2.3. Cohort Study Trial

**Figure 1:**
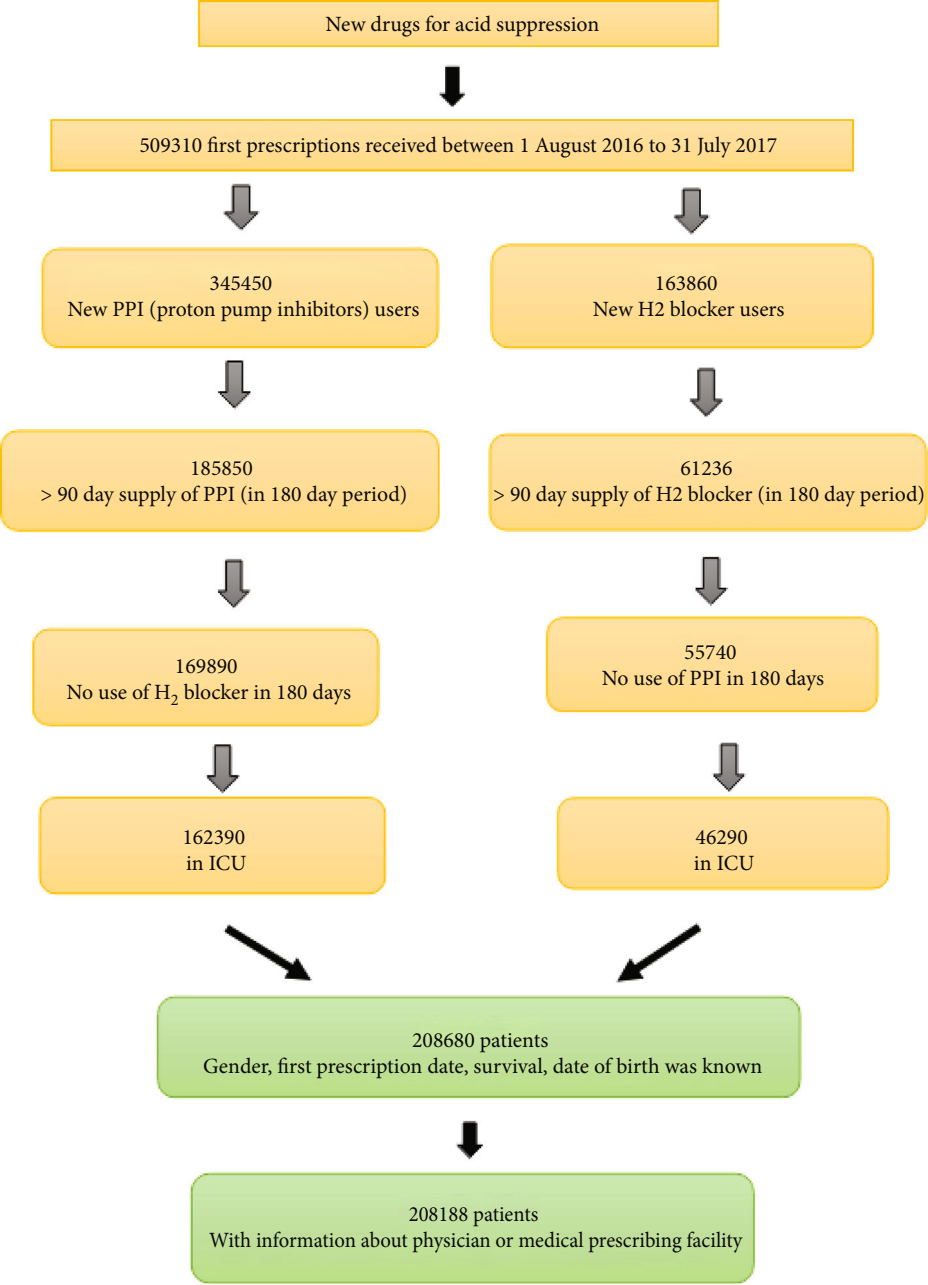
Cohort building flowchart.

A total of 208680 sepsis patients were divided into two groups, i.e., PPI user and H_2_ blocker user groups. A complete information about age, gender, date of birth, race, and survival post 180 days of the drug prescription was carefully recorded. Some of the new patients from routine clinics were included after the recommendation of the physicians, who also have a history of H_2_ blockers or PPI using and fulfilling our inclusion criteria. Thus, we finally recruited 208188 sepsis patients admitted to the ICU with a pure record of the PPI and H_2_ blockers (Figure 1).

### 2.4. Data Source

The data were obtained from a multicentre cohort study, “China Critical Care Sepsis Trial (CCCST) database,” which enrolled patients for the treatment of gastric sepsis. The dataset included inpatient and outpatient data, healthcare information, demographic profiles, comorbidities, clinical encounters, surgeries, and procedures.

### 2.5. Possible Causes of the Mortality

The data of clinical trials were also evaluated based on causes of death as classified with the national death index on ICD-10 (international classification of diseases). The reasons for death were further categorized into external causes, circulatory system diseases, metabolism, nutrition and endocrine diseases, digestive system diseases, respiratory system diseases, neoplasms, genitourinary system, behavioral and mental disorders, parasitic and infectious diseases, nervous system diseases, and various other causes. Based on the significant causes of death, the cases were further divided into subcauses, thus showing statistical significance, which showed clear evidence of the relationship between PPI and any adverse events which could be a reason for the mortality. Subcauses of death included upper gastrointestinal cancer, chronic kidney disease, cardiovascular diseases, and Clostridium difficile infections.

### 2.6. Statistical Analyses

The data were compiled using with SPSS 19.0 statistical software. Values are presented as means ± SEM. The characteristics of the study group and outcome of events from the PPI- and H_2_ blocker-using groups were reported based on mean, number, standard deviation, and percentages as required. To get unbiased results, the data from the target trials were randomly assigned for PPI and H_2_ blocker groups. A high-dimensional approach developed by Schneeweiss was applied to select potential founders and cofounders (included in data domains) associated with the PPI and H_2_ blocker consumption. Predefined covariables and covariates were selected algorithmically to generate the propensity scores. An inverse treatment probability weight was applied to the cohort based on propensity scores, resulting in the weighted pseudocohort.

## 3. Results

### 3.1. Demographic Characteristics of the Cohort

A total of 208188 (204357 males and 3831 females) Chinese sepsis patients with a mean age of 65.10 years admitted in the ICU were retrospectively analyzed. Initially, patients were divided into two groups (PPI group and H_2_ blocker group). Complete demography, associated diseases, type of drugs used, type of PPI or H_2_ blockers, used and mortality rate were studied in both groups. In total, 162016 (56.97%) were using PPI and 46172 (23.79%) using H+ blockers. Gender-based analysis showed 159396 (96.28%) males and 2620 (4.01%) females, using PPI, while 44961 (95.29%) males and 1211 (4.11%) females were treated with H blockers. However, both groups had an equal duration of inpatient stay in hospital; the number of smokers and nonsmokers in both groups was also similar (Table 1).

**Figure 2:**
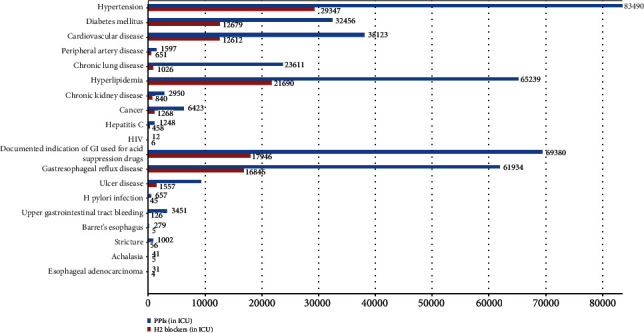
Comorbidities of sepsis patients at ICU.

Overall, analyses of the associated disease found that hyperlipidemia 86929 (41.98%) and GERD (gastroesophageal reflux disease) 78780 (38.12%) were the most occurring disease in both groups, followed by hypertension 112837 (26.11%), diabetes mellitus 45135 (23.12%), cardiovascular disease 50735 (22.34%), and chronic lung disease 24637 (12.54%). However, no significant difference was found among both groups (Table 1). In addition, the incidents of chronic kidney disease were detected in a total of 3790 (2.12%); several other diseases, including hepatitis C, HIV, H. pylori infection, achalasia, peripheral artery disease, and GIT cancers, were also detected in patients (Table 2 and Figure 2).

The stations 79281 (38.76%), ACE (angiotensin-converting enzyme) inhibitors 72219 (37.43%) and NSAIDs (nonsteroidal anti-inflammatory drugs) 43105 (27.58%) were the drugs other than PPI and H blockers also used by the patients (Table 3). However, no difference was seen in both groups.

**Figure 3:**
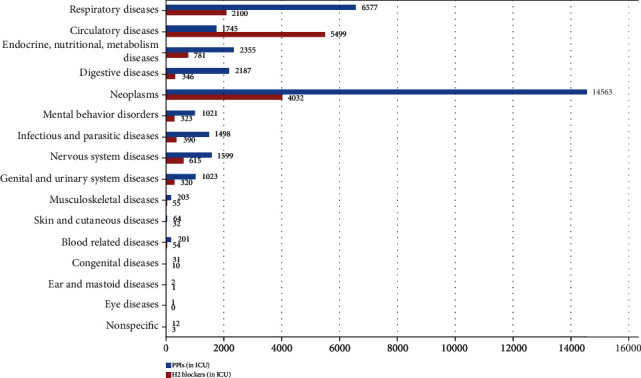
Causes of mortality in users of PPIs and H_2_ blockers.

In addition, we calculated the frequency of mortality in ICU patients. Data showed that circulatory diseases 7244 (12.23%), neoplasms 18595 (9.34%), and respiratory diseases 8677 (4.81%) were the top causes of mortality in patients using excessive PPIs and H_2_ blockers (Table 4 and Figure 3).

### 3.2. Commonly Used Proton Pump Inhibitors and H_2_ Blockers and Their Associated Adverse Effects

Next, we analyzed the efficacy of commonly used PPIs and H_2_ blockers; analyses showed that 78578 (62.17%) were prescribed to take omeprazole 20 mg once a day, lansoprazole 20 mg once a day to 31003 (17.88%), and pantoprazole 20 mg once a day to 14967 (9.27%) patients, while the H_2_ blockers ranitidine (150 mg/twice daily), cimetidine (200 mg/twice daily), and famotidine (20 mg/twice daily) were recommended for 35986 (71.34%), 6904 (16.59%), and 1795 (4.98%) patients, respectively (Table 5).

As a result of excessive use of these drugs (PPI and H blockers), 10.57% (CI: 10.02-12.97) in the PPI group while 7.67% (CI: 7.04-9.96) patients in the H_2_ blocker group reported acute kidney injury (*P* < 0.001), while chronic kidney disease was reported in 11.94% (CI: 9.56-14.86) and 8.56% (CI: 7.02-10.11) in the PPI and H_2_ blocker groups (*P* < 0.001), respectively. Transportation-related deaths (*P* = 0.23) and peptic ulcer disease-related deaths (*P* = 0.63) were also detected in both groups, but without significant differences (Table 6).

### 3.3. Mortality Associated with Excessive Use of PPI

For excessive use of PPI and H_2_ blockers has been associated with several secondary diseases, we further determined the possibility and rate of mortality associated with the extensive use of PPI and H blockers. Overall, deaths of 36.87% (CI: 36.23 to 39.45) and 31.96% (CI: 20.58-34.19) were associated with the patients using PPI and H_2_ blockers. Specifically, PPI-associated sepsis with chronic kidney disease 21.36% (CI: 9.34 to 23.23), circulatory diseases 16.34% (CI: 5.78 to 23.45), genitourinary system disorder, 5.23% (CI: 3.69 to 8.89), infectious and parasitic disease 4.17% (CI: 1.44 to 7.49), and respiratory diseases 2.16% (CI: -4.56 to 8.02) were the top causes of the mortality in the studied population. Esophageal adenocarcinoma, neoplasms, and GI cancers were also associated with the excessive use of PPI and H_2_ blockers. They are also causes of mortality in other patients (Table 7).

## 4. Discussion

The inappropriate prescriptions of the PPIs may increase the risk of unavoidable reactions, drug interactions, and hospitalization period [23]. It has been noticed that PPIs using frequency usually extends beyond the advised guidelines [24]. The PPIs are misconceptionally considered a safe drug even prescribed to the children [25]. Since 1990, several observational studies have pointed out some serious concerns related to patients' health, such as the fragility of the bones and fractures, acute interstitial nephritis, and acute kidney injury leading to chronic kidney disease [16, 26–29]. So far, no comprehensive analysis has reported the association of extensive use of PPIs and H_2_ blockers in sepsis patients in ICU and mortality rate. Here, for the first time, we performed a comprehensive analysis using a large population size to determine the association of PPI consumption in sepsis patients with CKD and other associated diseases.

Previously, an association between PPI consumption and acute kidney injury [15] and chronic kidney disease has been reported, suggesting a 20-50% increased risk of CKD. To reduce the risk of the PPI, it is highly recommended that PPI and H_2_ blocker therapy be strictly adopted for a limited time or till achieving minimum to moderate required outcomes. Alternative therapeutic approaches such as natural drugs or nutritional care would also be adopted to achieve the required goal. However, it has also been reported that 25% long-term PPI users showed no symptoms till discontinuing prescription [30]. Unfortunately, no latest stats are available; thus, it is strongly advised to reduce or avoid the unnecessary use of the PPI and H blockers. In the current study, we found that extensive use of PPIs and H_2_ blockers equally impacted the patients' health, causing severe cardiac problems, respiratory problems, acute kidney injury, and chronic kidney disease [31]. Several other conditions have also been reported in our cohort study. In short, we mainly focused on the association of PPI and H_2_ blockers with causes of mortality in the studied population. The retrospective data were collected from the China Critical Care Sepsis Trial (CCCST) database [32]. We found that 43.34% of deaths (95% CI 25.12 to 62.02) per 1000 patients were associated with the PPI and H_2_ blockers' users [33].

Interestingly, sepsis coupled with chronic kidney disease in ICU patients was the top contributor to mortality, followed by circulatory diseases, nervous system and mental disorders, genitourinary system disorder, and infectious and parasitic disease. Some of the real information regarding baseline health factors, drug use, or diseases may not correctly describe the patients. To avoid the involvement of other factors and to increase the reliability of our data, we obtained all possible information regarding pre/during hospitalization treatments and different non-PPI-associated diseases [34]. It has been suggested that PPI may be a significant cause of CKD [35], which is a bit contradictory to our findings. Interesting, in our study, CKD was not found among the top associated diseases with PPI and H_2_ blockers' excessive consumption, but was seen as the top associated cause of mortality in current patients [36]. Our study has some limitations such as the whole test design and implementation conditions are demanding, strictly controlled, and difficult, which is sometimes difficult to achieve in practical work [37]. Constrained by the scope of application of intervention measures, the selected research objects were not representative enough, which will affect the inference of experimental results to the whole in varying degrees. The study population is large and the follow-up time is long, so the compliance was also difficult, which affects the evaluation of experimental effect.

## 5. Conclusion

We demonstrate a significant association of PPI and H_2_ blockers with specific mortality causes in septic patients, such as chronic kidney disease, cardiovascular diseases, and upper gastrointestinal cancer. Individually, both PPI and H_2_ blockers did not show any significant difference in the impact of risk factors for other diseases and mortality. Since the prevalence of the PPI and H_2_ blockers is exceptionally high and poses a severe risk to public health, it is advised that the duration and doses of therapy must be reduced. Moreover, further researches must be performed to find probable solutions and alternative treatments. The characteristics of these drugs could be explored, especially the strong and long-lasting inhibition of gastric acid secretion than the anticholinergic drugs, short course of treatment for ulcer, high healing rate, and relatively few adverse reactions. Special attention should be paid to the serious consequences caused by improper use.

## Figures and Tables

**Table 1:** Basic information of experimental patients.

Characteristic		Total (%)	PPIs (in ICU) (%)	H_2_ blockers (in ICU) (%)	*P* value
Total number		208188	162016 (56.97)	46172 (23.79)	0.013
Mean age (SD) (years)		65.10 (12.25)	64.29 (11.39)	63.92 (10.11)	0.37
Male		204357 (96.11)	159396 (96.28)	44961 (95.29)	0.014
Female		3831 (4.07)	2620 (4.01)	1211 (4.11)	0.019
Admission in hospital in the last one year		18800 (8.81)	15328 (8.78)	3472 (8.83)	0.008
Length of hospital stay inpatients (IQR) median in days		7 (4 to 14)	7 (5 to 15)	7 (4 to 14)	0.17
No. of outpatient visits (IQR)		5 (2 to 11)	5 (2 to 11)	5 (2 to 11)	0.52
Smoking	Current	37263 (19.11)	26387 (18.39)	10876 (21.76)	0.009
	Former	37732 (20.09)	28967 (20.87)	8765 (19.38)	0.011
	Never	115325 (57.96)	87229 (56.03)	28096 (58.96)	<0.001

**Table 2:** Associated diseases in patients using PPI and H_2_ blockers extensively.

Characteristic	Total (%)	PPIs (in ICU) (%)	H_2_ blockers (in ICU) (%)	*P* value
Hypertension	112837 (26.11)	83490 (27.12)	29347 (25.38)	0.023
Diabetes mellitus	45135 (23.12)	32456 (22.45)	12679 (21.39)	0.19
Cardiovascular disease	50735 (22.34)	38123 (24.12)	12612 (23.19)	0.008
Peripheral artery disease	2248 (1.35)	1597 (1.12)	651 (1.59)	0.021
Chronic lung disease	24637 (12.54)	23611 (12.38)	1026 (12.67)	0.017
Hyperlipidemia	86929 (41.98)	65239 (41.59)	21690 (42.59)	0.005
Chronic kidney disease	3790 (2.12)	2950 (2.17)	840 (2.68)	0.018
Cancer	7691 (4.11)	6423 (4.12)	1268 (4.07)	0.17
Hepatitis C	1706 (0.87)	1248 (0.72)	458 (0.78)	0.102
HIV	18 (0.02)	12 (0.03)	6 (0.01)	0.002
Documented indication of GI used for acid suppression drugs	87326 (39.16)	69380 (48.11)	17946 (36.12)	<0.001
Gastroesophageal reflux disease	78780 (38.12)	61934 (40.12)	16846 (34.56)	0.005
Ulcer disease	10903 (6.11)	9346 (7.12)	1557 (5.13)	0.39
H. pylori infection	702 (0.34)	657 (0.41)	45 (0.17)	0.023
Upper gastrointestinal tract bleeding	3367 (1.61)	3451 (1.81)	126 (1.49)	<0.001
Barrett's esophagus	284 (0.27)	279 (0.31)	5 (0.01)	0.28
Stricture	1058 (0.61)	1002 (0.71)	56 (0.17)	0.019
Achalasia	46 (0.02)	41 (0.03)	5 (0.01)	0.05
Esophageal adenocarcinoma	35 (0.01)	31 (0.01)	4 (0.01)	0.004

**Table 3:** Use of other drugs among sepsis patients.

Characteristic	Total (%)	PPIs (in ICU) (%)	H_2_ blockers (in ICU) (%)	*P* value
ACE (angiotensin-converting enzyme) inhibitors	72219 (37.43)	59234 (38.34)	12985 (37.11)	0.056
NSAIDs (nonsteroidal anti-inflammatory drugs)	43105 (27.58)	27880 (25.55)	15225 (29.57)	0.07
Stations	79281 (38.76)	61255 (40.11)	18056 (37.23)	0.48
Mean systolic blood pressure (mmHg) (SD)	135.46 (19.07)	134.25 (19.11)	136.37 (19.05)	<0.001
Mean diastolic blood pressure (mmHg) (SD)	75.23 (11.29)	76.23 (11.45)	75.11 (11.12)	0.21
Mean GFR (glomerular filtration rate) (ml/min/1.73 m^2^) (SD)	73.56 (20.11)	72.66 (21.11)	74.87 (19.39)	0.014
Median high-density lipoprotein (mg/dl) (IQR)	41.77 (35.00-50.00)	41.33 (35.00-50.00)	42.87 (35.00-50.00)	0.46
Median low-density lipoprotein (mg/dl), (IQR)	107.22 (85.0-129.1)	106.66 (45.0-129.0)	108.56 (87.0-129.0)	0.002
Median (IQR) HbA1C	6.3 (5.1-6.1)	6.2 (5.5-6.9)	6.4 (5.5-7.1)	<0.001

**Table 4:** Difference in causes of mortality between PPIs and H_2_ blockers.

Characteristic	Total (%)	PPIs (in ICU) (%)	H_2_ blockers (in ICU) (%)	*P* value
Respiratory diseases	8677 (4.81)	6577 (4.72)	2100 (4.98)	0.024
Circulatory diseases	7244 (12.23)	1745 (12.99)	5499 (11.36)	<0.001
Endocrine, nutritional, metabolism diseases	3136 (1.76)	2355 (1.66)	781 (1.98)	0.29
Digestive diseases	2533 (1.22)	2187 (1.34)	346 (1.02)	0.455
Neoplasms	18595 (9.34)	14563 (9.01)	4032 (9.58)	0.009
Mental behavior disorders	1344 (1.34)	1021 (1.76)	323 (1.05)	0.015
Infectious and parasitic diseases	1888 (0.93)	1498 (1.02)	390 (0.87)	0.27
Nervous system diseases	2214 (1.21)	1599 (1.11)	615 (1.38)	0.319
Genital and urinary system diseases	1343 (1.87)	1023 (1.98)	320 (1.71)	0.007
Musculoskeletal diseases	258 (0.16)	203 (0.15)	55 (0.18)	<0.001
Skin and cutaneous diseases	96 (0.05)	64 (0.08)	32 (0.03)	0.013
Blood-related diseases	255 (0.09)	201 (0.12)	54 (0.07)	0.19
Congenital diseases	41 (0.01)	31 (0.01)	10 (0.01)	0.014
Ear and mastoid diseases	3 (0.00)	2 (0.00)	1 (0.00)	0.32
Eye diseases	1 (0.00)	1 (0.00)	0 (0.00)	0.029
Nonspecific	15 (0.01)	12 (0.01)	3 (0.01)	<0.001

**Table 5:** Top proton pump inhibitors and H_2_ blockers, used for prescription.

Ranking	PPI	*N* (%)	H_2_ blockers	*N* (%)	*P* value
1	Omeprazole 20 mg once a day	78578 (62.17)	Ranitidine (150 mg twice a day)	35986 (71.34)	0.014
2	Lansoprazole 20 mg once a day	31003 (17.88)	Cimetidine (200 mg twice daily)	6904 (16.59)	0.008
3	Pantoprazole 20 mg once a day	14967 (9.27)	Famotidine (20 mg twice a day)	1795 (4.98)	0.023

**Table 6:** Outcome controls: positive and negative.

Outcomes	Events per 100 (95% CI)		Excess (95% CI)	Hazard ratio	
	PPI	H_2_ blockers		Cox	Fine and gray
Acute kidney injury^∗^	10.57 (10.02-12.97)	7.67 (7.04-9.96)	13.07 (1.08 to 27.45)	1.07 (1.01-1.35)	1.15 (1.06-1.23)
Chronic kidney disease^∗^	11.94 (9.56-14.86)	8.56 (7.02-10.11)	14.97 (1.05 to 27.97)	1.05 (1.05-1.56)	1.06 (1.86-1.56)
Transportation-related death^∗∗^	0.31 (0.24-0.41)	0.37 (0.18-0.45)	-0.31 (-2.98 to 2.67)	0.95 (0.43-2.38)	0.93 (0.41-2.71)
Peptic ulcer disease-related death^∗∗∗^	0.03 (0.01-0.05)	0.07 (0.03-0.12)	-0.41 (-2.19 to 0.18)	0.41 (0.17-1.79)	0.38 (0.12-1.65)

^∗^ *P* < 0.001: positive control defined by ICD-9 584. ^∗∗^*P* > 0.1: negative outcome defined by ICD-10 V00-V99. ^∗∗∗^*P* > 0.5: negative outcome defined by CD-10 K211, K226, K20, and K250-K289.

**Table 7:** Causes of death in association with PPI (proton pump inhibitor) in 2 years of follow-up.

Cause of death	Rate per 100 (95%)		Burden per 1000 (95% CI)
	PPIs	H_2_ blockers	
All	36.87 (36.23 to 39.45)	31.96 (20.58-34.19)	43.34 (25.12 to 62.02)
Sepsis with chronic kidney disease	12.86 (11.08-14.58)	9.56 (8.07-10.23)	21.36 (9.34 to 23.23)
Circulatory diseases	12.98 (10.59-14.57)	10.45 (9.06-12.05)	16.34 (5.78 to 23.45)
Respiratory diseases	4.58 (3.98-5.03)	4.34 (4.02-4.98)	2.16 (-4.56 to 8.02)
Endocrine, metabolic, and nutritional disorders	1.21 (1.07-1.56)	1.19 (1.07-1.28)	-2.07 (-5.78 to 1.49)
Nervous system	1.55 (1.23-1.98)	1.04 (0.98-1.78)	2.08 (1.56 to 6.34)
Mental disorders	1.16 (1.08-1.20)	1.03 (0.98-1.17)	1.87 (1.65 to 2.95)
Genitourinary system	1.24 (1.02-1.67)	0.74 (0.56-0.98)	5.23 (3.69 to 8.89)
Infectious and parasitic disease	1.98 (1.56-2.01)	0.76 (0.56-0.98)	4.17 (1.44 to 7.49)
External causes	1.35 (1.03-1.78)	1.76 (1.56-1.98)	-3.56 (-10.39 to 1.48)
Esophageal adenocarcinoma	4.19 (3.77-4.96)	4.28 (4.02-4.68)	2.38 (1.56 to 4.02)
Barrett's esophagus	3.24 (2.53-5.06)	3.62 (2.37-4.93)	1.94 (1.06 to 3.92)
Neoplasms and GI cancers	1.27 (1.08-1.40)	1.63 (0.57-1.97)	1.87 (1.63 to 2.75)
Other causes	0.76 (0.56-0.98)	0.45 (0.32-0.78)	2.08 (-1.56 to 3.59)

## Data Availability

The raw data used for the current study will be available from the corresponding author upon reasonable request.
